# Pollen Analysis of Natural Honeys from the Central Region of Shanxi, North China

**DOI:** 10.1371/journal.pone.0049545

**Published:** 2012-11-21

**Authors:** Xiao-Yan Song, Yi-Feng Yao, Wu-De Yang

**Affiliations:** 1 College of Agronomy, Shanxi Agricultural University, Taigu, China; 2 State Key Laboratory of Systematic and Evolutionary Botany, Institute of Botany, Chinese Academy of Sciences, Beijing, China; University College London, United Kingdom

## Abstract

Based on qualitative and quantitative melissopalynological analyses, 19 Chinese honeys were classified by botanical origin to determine their floral sources. The honey samples were collected during 2010–2011 from the central region of Shanxi Province, North China. A diverse spectrum of 61 pollen types from 37 families was identified. Fourteen samples were classified as unifloral, whereas the remaining samples were multifloral. Bee-favoured families (occurring in more than 50% of the samples) included Caprifoliaceae (found in 10 samples), Laminaceae (10), Brassicaceae (12), Rosaceae (12), Moraceae (13), Rhamnaceae (15), Asteraceae (17), and Fabaceae (19). In the unifloral honeys, the predominant pollen types were *Ziziphus jujuba* (in 5 samples), *Robinia pseudoacacia* (3), *Vitex negundo* var. *heterophylla* (2), *Sophora japonica* (1), *Ailanthus altissima* (1), Asteraceae type (1), and Fabaceae type (1). The absolute pollen count (i.e., the number of pollen grains per 10 g honey sample) suggested that 13 samples belonged to Group I (<20,000 pollen grains), 4 to Group II (20,000–100,000), and 2 to Group III (100,000–500,000). The dominance of unifloral honeys without toxic pollen grains and the low value of the HDE/P ratio (i.e., honey dew elements/pollen grains from nectariferous plants) indicated that the honey samples are of good quality and suitable for human consumption.

## Introduction

Honey is naturally produced by honeybees from the nectar of plants. It is widely consumed as a health food product all over the world, but adulteration and the false labelling of honey are common problems in many countries [1]. In this context, melissopalynology plays an important role in ascertaining the botanical and geographical origins of honey by studying the pollen contained in the honey [1]–[6].

Shanxi Province is regarded as a rich source of honey in North China. The province’s great floristic diversity includes more than 80 families, 200 genera, and 600 species of nectar plants [7]. Beekeeping in Shanxi has high social and economic value. Beekeeping activities in the province can provide approximately 3000–6000 tons of commercial honey, 20–40 tons of royal jelly, and 75–150 tons of bee pollen each year [8]. These products are gaining increasing importance as they improve the socioeconomic situation of the people of Shanxi.

Although several melissopalynological studies have been conducted in China [9]–[17], most of these studies were based on qualitative analyses. Qualitative and quantitative melissopalynological analyses of Shanxi honeys are not yet available. Such analyses have not been conducted because of a lack of research on the botanical aspects of the honeys. The beekeepers do not know all the important nectar plants contributing to honey production. For this reason, the honey is sometimes mislabelled. Based on pollen analysis, this paper aims to determine the botanical characterisation of honeys from the central region of Shanxi for the first time and to provide a useful guide to beekeeping in this region.

## Materials and Methods

### Ethics Statement

No specific permits were required for the described field studies. The sampling sites are not protected in any way and the field studies did not involve endangered or protected species.

### Honey Sampling and Pollen Analysis

**Figure 1:**
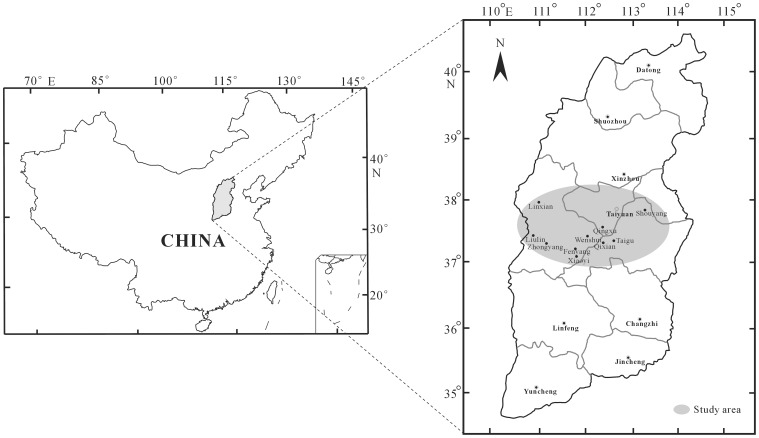
Location map showing the study area. (Left) map showing the position of Shanxi in China, (Right) map showing the sampling sites in the central region of Shanxi.

Nineteen natural honey samples (Table 1), produced primarily by *Apis mellifera* and *Apis cerana cerana*, were collected from nine Counties (Fig. 1) in the central region of Shanxi from April through September, 2010–2011.

**Table 1:** List of honey samples examined.

Sample No.	Locality	Latitude & Longitude	Time of collection
H1	Liulin County	37°26′ N, 110°52′ E	August 2011
H2	Zhongyang County	37°21′ N, 111°11′ E	August 2011
H3	Shouyang County	37°53′ N, 113°11′ E	August 2011
H4	Chengzhao Village, Qixian County	37°22′ N, 112°15′ E	May 2011
H5	Chengzhao Village, Qixian County	37°22′ N, 112°15′ E	June 2010
H6	Wangjiabao Village, Wenshui County	37°22′ N, 112°13′ E	August 2010
H7	Wangjiabao Village, Wenshui County	37°22′ N, 112°13′ E	April 2011
H8	Daxiang Village, Wenshui County	37°25′ N, 112°10′ E	May 2010
H9	Daxiang Village, Wenshui County	37°25′ N, 112°10′ E	May 2010
H10	Yian Village, Fenyang County	37°15′ N, 111°50′ E	April 2011
H11	Yian Village, Fenyang County	37°15′ N, 111°50′ E	September 2010
H12	Xiyangcheng Village, Fenyang County	37°12′ N, 111°46′ E	July–August 2010
H13	Linxian County	37°57′ N, 110°58′ E	June 2010
H14	Yulin Village, Qixian County	37°21′ N, 112°26′ E	May 2011
H15	Taigu County	37°25′ N, 112°32′ E	June 2010
H16	Taigu County	37°25′ N, 112°32′ E	June 2010
H17	Qingxu County	37°37′ N, 112°21′ E	July 2010
H18	Qingxu County	37°37′ N, 112°21′ E	April 2011
H19′	Qingxu County	37°37′ N, 112°21′ E	April 2011

For pollen analysis, the method recommended by the International Commission for Bee Botany [2] was adopted. Ten grams of each honey was dissolved in 20 ml of warm water (40°C). The solution was centrifuged for 10 min at 2500 r/min, the supernatant solution was decanted, and the sediments were collected into a conical tube and treated with an acetolysis mixture (acetic anhydride : conc. sulphuric acid = 9∶1 V/V) [18] for approximately 30 min at room temperature. After treatment with the acetolysis mixture, the sediments were rinsed with distilled water, centrifuged for 5 min at 2500 r/min, and preserved for study.

**Figure 2:**
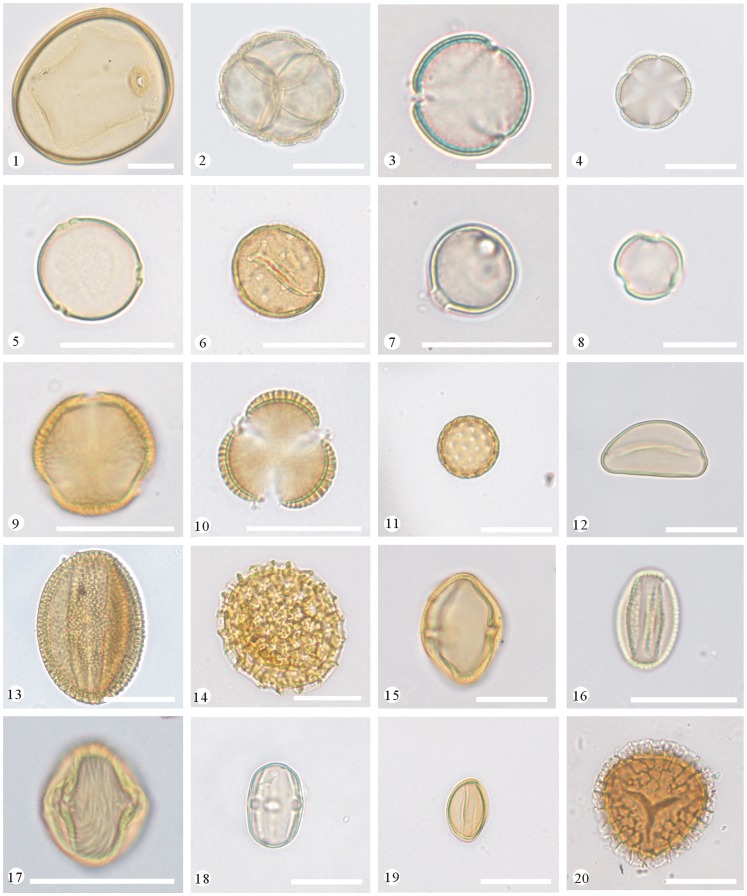
Photomicrographs of selected pollen grains recovered from the honey samples. 1. Poaceae type, 2. *Catalpa ovata* (Bignoniaceae), 3. Ranunculaceae type, 4. Laminaceae type, 5. *Humulus* sp. (Moraceae), 6. *Juglans regia* (Juglandaceae), 7. *Glycine max* (Fabaceae), 8. *Sophora japonica* (Fabaceae), 9. *Ailanthus altissima* (Simaroubaceae), 10. Brassicaceae type, 11. Chenopodiaceae type, 12. *Allium* sp. (Liliaceae), 13. *Fagopyrum esculentum* (Polygonaceae), 14. Polygonaceae type, 15. *Rhus* sp. (Anacardiaceae), 16. *Salix* sp. (Salicaceae), 17. *Prunus* sp. (Rosaceae), 18. *Melilotus suareolens* (Fabaceae), 19. *Vitex negundo* var. *heterophylla* (Verbenaceae), 20. *Lycopodium* sp. (Lycopodiaceae) (Scale bar = 20 µm for Nos. 1, 2, 4–7, 9–20, = 10 µm for Nos. 3, 8).

**Figure 3:**
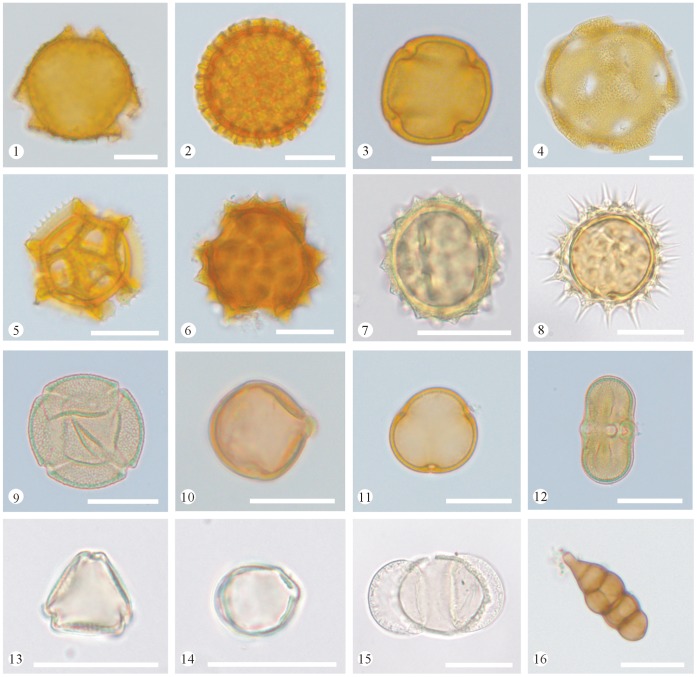
Photomicrographs of selected pollen grains recovered from the honey samples (continued). 1. *Lonicera maackii* (Caprifoliaceae), 2. *Syringa* sp. (Oleaceae), 3. Meliaceae type, 4. *Convolvulus arvensis* (Convolvulaceae), 5. *Taraxacum mongolicum* (Asteraceae), 6–8. Asteraceae type, 9. Polygonaceae type, 10. *Robinia pseudoacacia* (Fabaceae), 11. Unidentified pollen, 12. Apiaceae type, 13–14. *Ziziphus jujuba* (Rhamnaceae), 15. *Pinus* sp. (Pinaceae), 16. Fungi spore (Scale bar = 20 µm).

To analyse the pollen content of the honey samples, two slides were prepared from each sample and photographed under a Leica DM2500 light microscope. Pollen types were identified by comparison with reference slides of pollen collected directly from the plants in the study area. In addition, selected palynological literature and monographs [19]–[21] were used. Photomicrographs of different types of pollen grains recovered from the honey samples are shown in Figs. 2 and 3.

For quantification of the pollen types, at least 500 pollen grains were counted from each sample. The percentage frequency of the pollen taxa in all the samples was calculated. The types of pollen were allocated to one of four frequency classes: (i) predominant pollen types (>45% of the total pollen grains counted); (ii) secondary pollen types (16%–45%); (iii) important minor pollen types (3%–15%); and (iv) minor pollen types (<3%). The honey sample was characterised as unifloral if it contained a predominant pollen type. Otherwise, it was considered multifloral.

The absolute pollen counts (APC) of the honey sample (i.e., the number of pollen grains per 10 g honey) were calculated with a haemocytometer [22]. Pollen grains were counted under a microscope at 100× magnification over a haemocytometer (counting chamber). The chamber is 0.1 mm high and has 25 medium squares of 0.04 mm^2^ each, which are subdivided into 16 small squares of 0.0025 mm^2^ each. This means a volume of 0.1 µl in the chamber, 0.004 µl in each medium square and 0.00025 µl in each small one. For each sample, we counted the pollen grains of five medium squares at the center, left and right corners at the top and bottom of the chamber, which was repeated for making 100 individual observations. Based on the average number of 100 observations, the absolute pollen counts in the volume of 100 µl suspension with the pollen sediment contained in 10 g honey were calculated. The samples were classified into five groups as proposed by Louveaux et al. [2]: Group I (<20,000 pollen grains); Group II (20,000–100,000); Group III (100,000–500,000); Group IV (500,000–1,000,000); and Group V (>1,000,000).

To determine the frequency of honey dew elements (HDE), a HDE/P ratio was calculated for each honey. The honey dew elements were calculated by counting the number of honey dew elements (HDE) and dividing by the total frequency of pollen grains from nectariferous plants (P), following Louveaux et al. [2].

In addition, the ecological parameter, Shannon-Weaver diversity index, was used to calculate the pollen diversity in each sample [5], [23] according to the following equation:
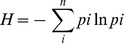



(H: Shannon-Weaver diversity index, pi: proportion of each pollen type i encountered in the sample, ln: natural logarithm).

## Results

A total of 61 pollen types belonging to 37 families were identified from 19 honey samples, including 56 melliferous pollen types (insect-pollinated) and 5 non-melliferous pollen types (wind-pollinated) (Tables 2, 3, 4, 5). Samples H1 and H11 showed the minimum (n = 7) and maximum (n = 22) number of plant taxa, respectively. Unidentified pollen grains were found in 6 samples (H4, H9, H12, H14, H15, and H16) at a low frequency (0.35–4.56%) (Tables 2, 3, 4, 5).

**Table 2:** Pollen types recovered from the honey samples and their frequency.

Pollen types	Honey sample no.																		
	H1	H2	H3	H4	H5	H6	H7	H8	H9	H10	H11	H12	H13	H14	H15	H16	H17	H18	H19
**Melliferous** **pollen**																			
**Anacardiaceae**																			
*Cotinus* sp.	1.00							5.66	1.07										
*Rhus* sp.					2.76														
**Apiaceae**																			
Apiaceae type		0.20					0.47									0.17			
**Araliaceae**																			
Araliaceae type			0.20			4.55													
**Asteraceae**																			
*Artemisia* sp.		0.20			0.20			0.20	0.27	1.02		0.77	0.39	0.38				0.16	1.34
Asteraceae type			1.60						0.13		16.22	2.13	1.55	48.85		8.38		0.48	
*Taraxacum* *mongolicum*	0.40			8.36	0.39	3.23	6.61			5.26						9.60			0.95
**Bignoniaceae**																			
*Catalpa ovata*										23.77	0.62								
*Markhamia* sp.									0.54						0.35	0.35	0.20		
**Brassicaceae**																			
Brassicaceae type				9.25	0.59	8.21	3.78		0.27	10.19	9.20	10.25	0.39	0.77		0.35			2.48
**Caprifoliaceae**																			
*Lonicera maackii*				0.15	0.39		0.16		0.94		0.47					0.52	2.60	0.63	1.53
*Sambucus* sp.										3.74									
**Convolvulaceae**																			
*Convolvulus arvensis*		0.20		6.12										0.19		1.22			0.38
**Euphorbiaceae**																			
Euphorbiaceae type															0.70				
**Fabaceae**																			
Fabaceae type				22.54	11.02	9.38	60.63	21.82	5.90	3.23	7.02			8.05		2.97			
*Gleditsia* sp.																0.52			
*Glycine max*												7.16		0.19					
*Glycyrrhiza* sp.								7.88		0.34									
*Medicago* sp.	0.20	0.40	0.40						5.90	3.06	0.31	0.19			2.26			0.16	
*Melilotus* *suareolens*			0.40			1.61		6.87	0.40	0.17	4.37	11.80			0.35				

**Table 3:** Pollen types recovered from the honey samples and their frequency (continued).

Pollen types	Honey sample no.																		
	H1	H2	H3	H4	H5	H6	H7	H8	H9	H10	H11	H12	H13	H14	H15	H16	H17	H18	H19
*Robinia pseudoacacia*	0.40	72.40							14.75	18.85	3.90	1.16	2.91	1.53	3.48		15.20	61.01	69.08
*Sophora japonica*	0.60	12.00	6.80			20.67		7.88	40.88	2.04	9.05	0.58	1.94		18.47	0.17	77.40	28.21	13.74
*Sophora viccifolia*		0.20	0.20		0.20					4.41	0.31			4.21			2.80		
*Vicia* sp.			0.20																
**Fagaceae**																			
*Lithocarpus* sp.												4.26							
**Gentianaceae**																			
Gentianaceae type					0.39		0.16								0.87	2.79			
**Juglandaceae**																			
*Juglans regia*			0.20																
**Laminaceae**																			
Laminaceae type			0.20	0.60	0.59				1.47			0.19	0.19	0.77	0.35	1.05	0.20		
**Liliaceae**																			
*Allium* sp.				0.90			5.51							3.64		0.17			
*Lilium* sp.										3.06	0.16			0.19		0.35			0.19
**Magnoliaceae**																			
*Magnolia* sp.						0.73	0.31								0.17				
**Malvaceae**																			
Malvaceae type								0.40											
**Meliaceae**																			
Meliaceae type				0.15								0.58			0.17				
**Moraceae**																			
*Humulus* sp.		0.40	1.80	2.99	0.59		0.16	0.20		0.17	0.31	3.48		0.19	0.17		0.20		1.91
**Myricaceae**																			
*Myrica* sp.						0.15													
**Oleaceae**																			
*Syringa* sp.		0.20	7.60	1.04										2.11		0.87		0.16	0.76
**Polygonaceae**																			
*Fagopyrum esculentum*			0.20			0.15							0.19			0.17			
*Polygonum* sp.		0.40	0.20																
*Rheum* sp.											0.16								
**Rhamnaceae**																			

**Table 4:** Pollen types recovered from the honey samples and their frequency (continued).

Pollen types	Honey sample no.																		
	H1	H2	H3	H4	H5	H6	H7	H8	H9	H10	H11	H12	H13	H14	H15	H16	H17	H18	H19
*Ziziphus jujuba*	97.00	5.00	6.20		4.53	50.44		48.89	21.18	0.85	31.20	10.25	92.44	0.96	1.92	64.75			1.91
**Rosaceae**																			
*Exochodra* sp.												41.78							
*Malus* sp.	0.40	6.80	0.60	4.33															
*Prunus* sp.		1.00					1.73								7.14		1.40	7.13	5.15
*Pyrus* sp.		0.20		1.64					0.67										
*Rosa* sp.			0.20																
Rosaceae type				5.22										25.48	0.70				
**Salicaceae**																			
*Salix* sp.				3.13			20.00		0.13					0.38	0.17				0.38
**Sapindaceae**																			
*Koelreuteria paniculata*											2.34								
**Scrophulariaceae**																			
*Paulownia sp.*				28.21														2.06	
Scrophulariaceae type					17.13														
**Simaroubaceae**																			
*Ailanthus altissima*					61.02						3.74								
**Solanaceae**																			
Solanaceae type				1.34						0.51									
**Tiliaceae**																			
*Tilia* sp.											0.16								
**Ulmaceae**																			
*Ulmus* sp.		0.40																	
**Verbenaceae**																			
Verbenaceae type			3.20																
*Vitex negundo* var.*heterophylla*			63.60								9.20			0.57	62.37				

**Table 5:** Pollen types recovered from the honey samples and their frequency (continued).

Pollen types			Honey sample no.																					
			H1				H2	H3	H4	H5	H6	H7	H8	H9	H10	H11	H12	H13	H14	H15	H16	H17	H18	H19
**Non-melliferous pollen**																								
**Chenopodiaceae**																								
Cheonpodiaceae type								2.60		0.20	0.29					0.16	0.19				4.54			0.19
**Cyperaceae**																								
Cyperaceae type																0.16	0.58							
**Pinaceae**																								
*Pinus* sp.													0.20	0.67			0.19		0.38		0.17			
**Poaceae**																								
Poaceae type								3.60	0.90		0.59	0.47				0.16	0.58		0.57		0.52			
**Ranunculaceae**																								
Ranunculaceae type														0.27	19.35	0.78	1.16		0.19					
																			
**Total number of pollen types**	7			15				20	18	14	12	12	10	18	17	22	20	8	21	17	21	8	9	14
**Number of melliferous pollen types**	7			15				18	16	13	10	11	9	15	16	18	14	8	17	16	17	8	9	13
**No. of non-melliferous pollen types**	–			–				2	1	1	2	1	1	2	1	4	5	–	3	–	3	–	–	1
**Number of unknown pollen types**	–			–				–	1	–	–	–	–	1	–	–	1	–	1	1	1	–	–	–
**Unknown pollen frequency (%)**	–			–				–	3.13	–	–	–	–	4.56	–	–	2.71	–	0.38	0.35	0.35	–	–	–
**No. of melliferous pollen families**	5			10				11	14	11	8	11	6	10	9	13	9	6	12	12	13	6	6	11
**No. of non-melliferous pollen** **families**	–			–				2	1	1	2	1	1	1	1	4	5	–	3	–	3	–	–	1
**No. of plant families in each sample**	5			10				13	15	12	10	12	7	11	10	17	14	6	15	12	16	6	6	12
**HDE/P ratio**	0			0				0	0	0.014	0.009	0	0.036	0	0.005	0.011	0.008	0.025	0.006	0.016	0.017	0	0	0
**Shannon-Weaver diversity index**	0.18			1.03				1.48	2.21	1.28	1.52	1.26	1.49	1.79	2.18	2.18	2.01	0.38	1.62	1.31	1.42	0.78	1.01	1.19

**Figure 4:**
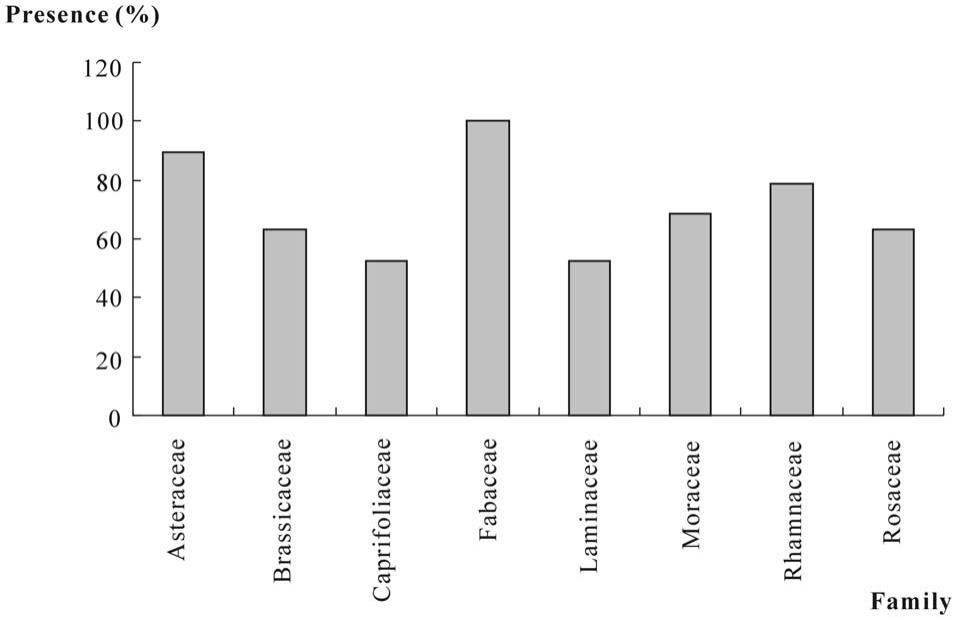
Families found in more than 50% of the honey samples. Caprifoliaceae and Laminaceae, found in 10 samples (52.63%); Brassicaceae and Rosaceae, found in 12 samples (63.16%); Moraceae, found in 13 samples (68.42%); Rhamnaceae, found in 15 samples (78.95%); Asteraceae, found in 17 samples (89.47%); Fabaceae, found in all samples (100%).

**Figure 5:**
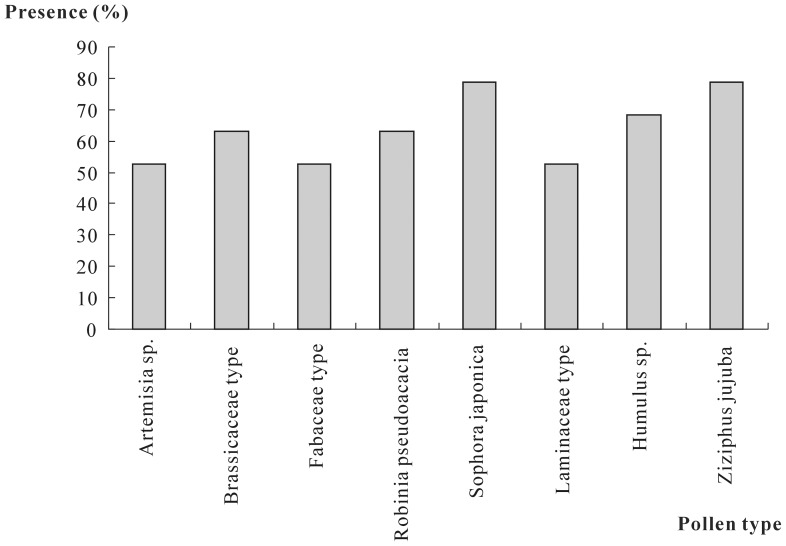
Pollen types found in more than 50% of the honey samples. *Artemisia* sp., Fabaceae type, and Laminaceae type, found in 10 samples (52.63%); Brassicaceae type and *Robinia pseudoacacia*, found in 12 samples (63.16%); *Humulus* sp., found in 13 samples (68.42%); *Sophora japonica* and *Ziziphus jujuba*, found in 15 samples (78.95%).

Families that occurred in more than 50% of the honey samples included Caprifoliaceae (found in 52.63% (n = 10) of the samples), Laminaceae (52.63%, n = 10), Brassicaceae (63.16%, n = 12), Rosaceae (63.16%, n = 12), Moraceae (68.42%, n = 13), Rhamnaceae (78.95%, n = 15), Asteraceae (89.47%, n = 17), and Fabaceae (100%, n = 19) (Fig. 4). Eight pollen types were found in more than one-half of the samples. These pollen types included *Artemisia* sp. (52.63%, n = 10), Fabaceae type (52.63%, n = 10), Laminaceae type (52.63%, n = 10), Brassicaceae type (63.16%, n = 12), *Robinia pseudoacacia* (63.16%, n = 12), *Humulus* sp. (68.42%, n = 13), *Sophora japonica* (78.95%, n = 15), and *Ziziphus jujuba* (78.95%, n = 15) (Fig. 5).

**Figure 6:**
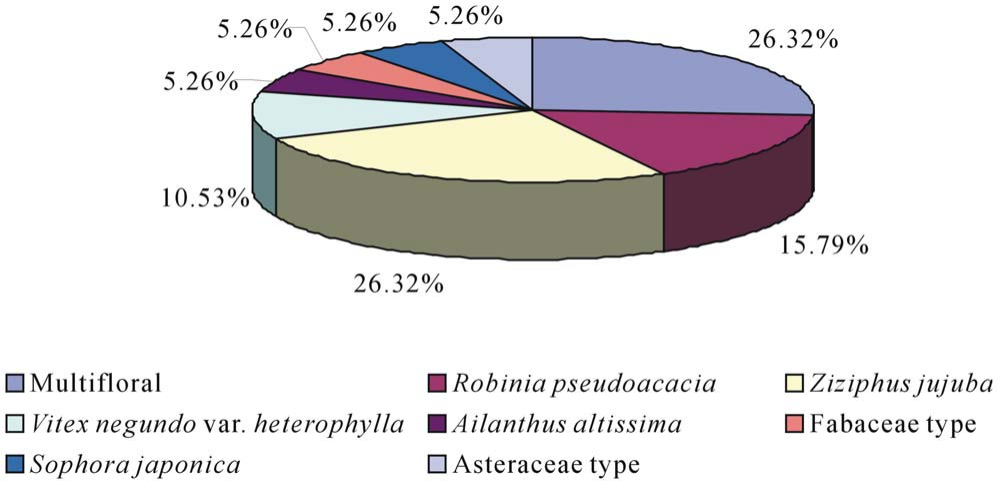
Botanical origin of honey samples from the central region of Shanxi. Of these samples, 5 honeys were multifloral (26.32%) and 14 were unifloral (73.68%): 5 of *Ziziphus jujuba* (26.32%), 3 of *Robinia pseudoacacia* (15.79%), 2 of *Vitex negundo* var. *heterophylla* (10.53%), 1 of *Ailanthus altissima* (5.26%), 1 of *Sophora japonica* (5.26%), 1 of Asteraceae type (5.26%), 1 of Fabaceae type (5.26%).

Of the 19 honey samples, 5 were classified as multifloral (26.32%) and 14 as unifloral (73.68%), represented by 7 predominant pollen types: *Ziziphus jujuba* (26.32%, n = 5), *Robinia pseudoacacia* (15.79%, n = 3), *Vitex negundo* var. *heterophylla* (10.53%, n = 2), *Sophora japonica* (5.26%, n = 1), *Ailanthus altissima* (5.26%, n = 1), Asteraceae type (5.26%, n = 1), and Fabaceae type (5.26%, n = 1) (Fig. 6).

**Figure 7:**
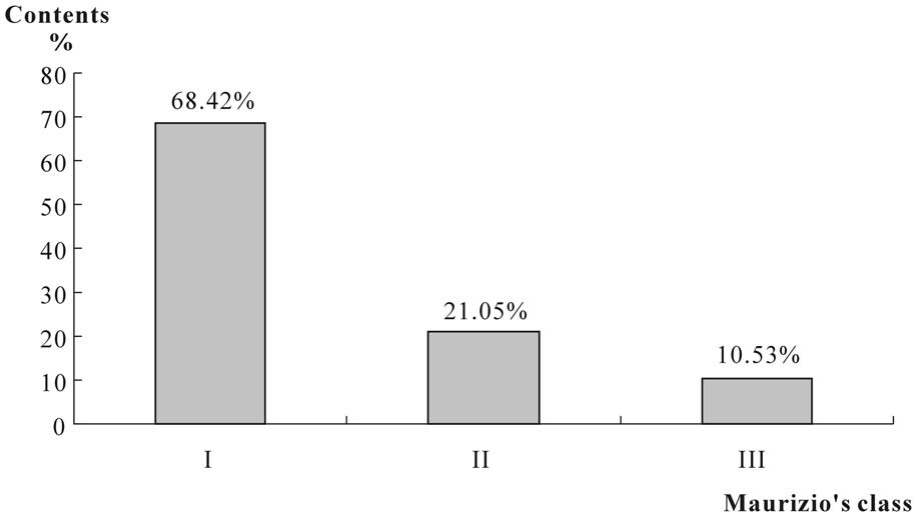
Distribution (%) of the honey samples according to Maurizio’s classes. Group I (<20,000 pollen grains per 10 g honey) found in 13 samples (68.42%), Group II (20,000–100,000 grains per 10 g honey) found in 4 samples (21.05%), Group III (100,000–500,000 grains per 10 g honey) found in 2 samples (10.53%).

Based on the absolute pollen count per 10 g of the honey samples, 68.42% (n = 13) of the samples were found to belong to Group I (actual values 2500–15,000 pollen grains), 21.05% (n = 4) to Group II (25,000–60,000), and 10.53% (n = 2) to Group III (120,000–142,500) (Table 6, Fig. 7).

**Table 6:** Pollen analytical data of honey samples from the central region of Shanxi, North China.

Honey sample	APC/10 g honey	Maurizio’s classes	Nature of honey
H1	142, 500	III	*Ziziphus jujuba* unifloral (97.00%)
H2	10, 000	I	*Robinia pseudoacacia* unifloral (72.40%) with *Sophora japonica* (12.00%), *Malus* sp. (6.80%), *Ziziphus jujuba* (5.00%)
H3	15, 000	I	*Vitex negundo* var. *heterophylla* unifloral (63.60%) with *Syringa* sp. (7.60%), *Sophora japonica* (6.80%), *Ziziphus jujuba* (6.20%)
H4	25, 000	II	Multifloral: Fabaceae type (22.54%), *Paulownia* sp. (28.21%), Brassicaceae type (9.25%), *Taraxacum mongolicum* (8.36%)
H5	2500	I	*Ailanthus altissima* unifloral (61.02%) with Scrophulariaceae type (17.13%), Fabaceae type (11.02%)
H6	52, 500	II	*Zizyphus jujuba* unifloral (50.44%) with *Sophora japonica* (20.67%), Fabaceae type (9.38%)
H7	2500	I	Fabaceae type unifloral (60.63%) with *Salix* sp. (20.00%), *Taraxacum mongolicum* (6.61%), *Allium* sp. (5.51%)
H8	5000	I	*Zizyphus jujuba* unifloral (48.89%) with Fabaceae type (21.82%), *Glycyrrhiza* sp. (7.88%), *Sophora japonica* (7.88%), *Melilotus suareolens* (6.87%), *Cotinus* sp. (5.66%)
H9	60, 000	II	Multifloral: *Sophora japonica* (40.88%), *Zizyphus jujuba* (21.18%), *Robinia pseudoacacia* (14.74%)
H10	7500	I	Multifloral: *Catalpa ovata* (23.77%), *Robinia pseudoacacia* (18.85%), Ranunculaceae type (19.35%), Brassicaceae type (10.19%), *Taraxacum mongolicum* (5.26%)
H11	12, 500	I	Multifloral: *Zizyphus jujuba* (31.20%), Asteraceae type (16.22%), *Vitex negundo* var. *heterophylla* (9.20%), Brassicaceae type (9.20%), *Sophora japonica* (9.05%), Fabaceae type (7.02%)
H12	7500	I	Multifloral: *Exochodra* sp. (41.78%), *Melilotus suareolens* (11.80%), Brassicaceae type (10.25%), *Zizyphus jujuba* (10.25%), *Glycine max* (7.16%)
H13	120, 000	III	*Ziziphus jujuba* unifloral (92.44%) with *Robinia pseudoacacia* (2.91%)
H14	5000	I	Asteraceae type unifloral (48.85%) with Rosaceae type (25.48%), Fabaceae type (8.05%)
H15	15, 000	I	*Vitex negundo* var. *heterophylla* unifloral (62.37%) with *Sophora japonica* (18.47%), *Prunus* sp. (7.14%)
H16	10, 000	I	*Zizyphus jujuba* (64.75%) unifloral with *Taraxacum mongolicum* (9.60%), Asteraceae type (8.38%)
H17	32, 500	II	*Sophora japonica* (77.40%) unifloral with *Robinia pseudoacacia* (15.20%)
H18	10, 000	I	*Robinia pseudoacacia* unifloral (61.01%) with *Sophora japonica* (28.21%), *Prunus* sp. (7.13%)
H19	10, 000	I	*Robinia pseudoacacia* unifloral (69.08%) with *Sophora japonica* (13.74%), *Prunus* sp (5.15%)

Honey dew elements were considered absent from the samples due to the low HDE/P values found (0–0.036) (Table 5). The Shannon–Weaver diversity index values of the multifloral honeys ranged from 1.79 to 2.21, whereas the unifloral honeys showed lower values, from 0.18 to 1.62 (Table 5).

## Discussion and Conclusion

Pollen is very important for honeybee nutrition [24], [25]. Honeybees collect pollen grains from entomophilous and anemophilous plants to obtain protein for their survival and reproduction [17], [26]. The bees frequently collect a wide variety of pollen types, but they generally concentrate on a few species [27], [28]. The present study provides new insights into the pollen composition of honey samples from the central region of Shanxi, North China. A total of 61 pollen types from 19 honeys produced by *Apis mellifera* and *Apis cerana cerana* were identified, including 56 entomophilous pollen types (e.g., crop plants: *Glycine max*, *Vicia* sp., *Fagopyrum esculentum*, fruit trees: *Prunus* sp., *Pyrus* sp.) and 5 anemophilous pollen types (e.g., Chenopodiaceae, Cyperaceae, Poaceae). The Shannon–Weaver diversity index shows high diversity of pollen types in 5 multifloral honeys with a range of 1.79 (sample H9) to 2.21 (sample H4). High values in samples H10, H11, and H12 indicates rich nectar and pollen sources in Fengyang County in April, July to September. While compared with the multifloral honeys, 14 unifloral honeys have lower values of diversity index, ranging from 0.18 (sample H1) to 1.62 (sample H14), which suggests the diversity of pollen types has great changes in honey samples collected from different localities.

In the local vegetation of central Shanxi, typical cultivated plants are represented by *Juglans regia*, *Prunus* spp., *Robinia pseudoacacia*, *Sophora japonica*, *Vitis vinifera*, and *Ziziphus jujuba*, whereas the dominant wild plants include *Hippophae rhamnoides*, *Vitex negundo* var. *heterophylla*, and *Ziziphus jujuba* var. *spinosa* [29]. In this study, seven predominant pollen types (i.e., *Ailanthus altissima*, Asteraceae type, Fabaceae type, *Robinia pseudoacacia*, *Sophora japonica*, *Vitex negundo* var. *heterophylla*, *Ziziphus jujuba*) were recorded in fourteen unifloral honeys. The local beekeepers usually know that the latter four types are major nectar plants in this region, but they may not know that the former three types can also be used as principal nectar sources by honeybees. Asteraceae and Fabaceae are two large families, comprising approximately 90 and 100 species, respectively, in Shanxi [29]. In addition to the major nectar plants, the plants frequently used by honeybees for foraging included *Catalpa ovata*, *Exochodra* sp., *Paulownia* sp., *Salix* sp., Scrophulariaceae type, and Rosaceae type. The analysis of the pollen content of the honey samples indicates that the local flora may be used as a source of good-quality honey.

The majority (73.68%) of the 19 honey samples were considered unifloral honeys because they contained a predominant pollen type (frequency >45%). The dominance of unifloral honeys without any toxic pollen grains and with scarce fungal elements suggests that most of the honey samples are of good quality and suitable for human consumption.
